# BRCA1 regulates the cancer stem cell fate of breast cancer cells in the context of hypoxia and histone deacetylase inhibitors

**DOI:** 10.1038/s41598-019-46210-y

**Published:** 2019-07-04

**Authors:** Hoon Kim, Qun Lin, Zhong Yun

**Affiliations:** 1 Department of Therapeutic Radiology, Yale School of Medicine, New Haven, CT 06510 USA; 2 Present Address: Oncology & Immunology Research Team II, LG Chem. LG Science Park, E8 30 Magokjungang 10-ro Gangseo-gu, Seoul, 07796 Korea

**Keywords:** Cancer stem cells, Breast cancer, Cancer microenvironment

## Abstract

Cancer cell stemness is essential for enabling malignant progression and clonal evolution. Cancer cell fate is likely determined by complex mechanisms involving both cell-intrinsic pathways and stress signals from tumor microenvironment. In this study, we examined the role of the tumor suppressor BRCA1 and hypoxia in the regulation of cancer cell stemness using genetically matched breast cancer cell lines. We have found that BRCA1, a multifunctional protein involved in DNA repair and epigenetic regulation, plays a critical role in the regulation of cancer stem cell (CSC)-like characteristics. Reconstitution of BRCA1 resulted in significant decrease of the CSC-like populations in breast cancer cells whereas down-regulation of BRCA1 resulted in significant increase of the CSC-like populations. Furthermore, the BRCA1-reconstituted tumor cells are more sensitive to the histone deacetylase (HDAC) inhibitor-induced loss of stemness than the BRCA1-deficient cells are. Surprisingly, hypoxia preferentially blocks HDAC inhibitor-induced differentiation of the BRCA1-reconstituted breast cancer cells. In light of the increasing numbers of clinical trials involving HDAC inhibitors in human cancers, our observations strongly suggest that the BRCA1 status and tumor hypoxia should be considered as potentially important clinical parameters that may affect the therapeutic efficacy of HDAC inhibitors.

## Introduction

Tumor cells, even cell lines *in vitro*, consist of mixed populations some of which are capable of tumor initiation and possess stem cell-like characteristics. These tumor-initiating cells (TICs), also interchangeably termed as cancer stem cells (CSC), are thought to be the major cause of therapy resistance and tumor recurrence^1^. The cell fate of tumor cells, just like that of normal stem or progenitor cells, is subject to tight regulations by intrinsic genetic and epigenetic factors, as well as by their niche microenvironment.

The tumor suppressor gene *BRCA1* is frequently mutated in human cancers including breast cancer, ovarian cancer and prostate cancer^2,3^. BRCA1 protein plays a critical role in error-free DNA repair and its mutation is associated with global chromosome instability and tumor formation^4–6^. BRCA1 has also been found to play an important role in chromatin remodeling and gene transcription, indicating that BRCA1 may have pleiotropic functions during tumor development^7–9^. Interestingly, BRCA1 has been shown to be required for differentiation of mammary stem/progenitor cells to luminal epithelial cells^10,11^, suggesting that BRCA1 constitutes an important intrinsic pathway involved in cell fate determination.

As an emerging concept, tumor microenvironment can potentially provide a unique niche for CSCs to survive and continuously propagate^12–14^. Increasing evidence shows that hypoxia, a condition of oxygen deficiency and a hallmark of tumor microenvironment (TME), up-regulates CSC-related genes, promotes self-renewal and suppresses cell differentiation^15,16^. A number of *in vitro* studies have shown that hypoxia or hypoxia-sensing pathways play a significant role in the maintenance of the CSC phenotype in breast cancer cells^17–23^. Hypoxia is also implicated in increased CSC-like populations in breast cancer xenografts treated by antiangiogenic agents^24^. We have recently found direct evidence that CSC-like population of breast cancer cells are significantly enriched in the hypoxic regions *in vivo*^25^. Interestingly, it has been shown that *BRCA1* transcription is strongly repressed under hypoxic conditions^26,27^, suggesting that inadequate BRCA1 expression and functions can be found in the hypoxic tumor microenvironment in solid tumors. These findings suggest that hypoxia and downregulation of BRCA1 could synergize to enhance and/or maintain stem cell characteristics of cancer cells.

In this study, we examined the role of BRCA1 in the regulation of breast cancer cell stemness. Reconstitution of BRCA1 expression in the BRCA1-mutated HCC1937 cells resulted in a decrease of the CSC-like populations. On the other hand, down-regulation of BRCA1 in SKBR3 breast cancer cells significantly increased the CSC-like populations. Hypoxia facilitated the enrichment of the CSC-like populations in both BRCA1-competent and BRCA1-deficient breast cancer cells. Furthermore, we found that the BRCA1-reconstituted tumor cells were more sensitive than the BRCA1-mutated cells to histone deacetylase (HDAC) inhibitor-induced differentiation. Interestingly, hypoxia significantly blocked HDAC inhibitor-induced differentiation, especially, of the BRCA1-competent breast cancer cells. Our data strongly suggest that BRCA1 does not only regulate cancer cell fate but also affect how cancer cells respond to tumor microenvironmental stresses and therapeutic drugs.

## Results

### BRCA1 suppresses cancer stem cell-like characteristics of human breast cancer cells

**Figure 1:**
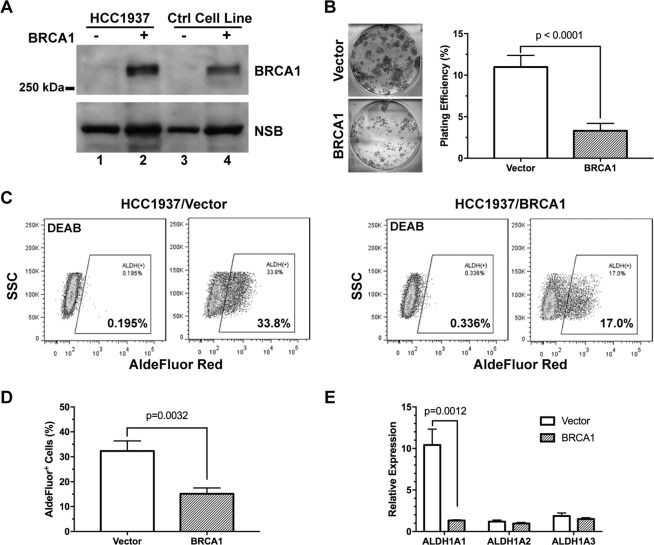
BRCA1 suppresses ALDH1A expression and activity in breast cancer cells. (**A**) Wild-type BRCA1 was reconstituted in HCC1937 human breast cancer cell line by retroviral transduction (Lane 2) with the empty vector as control (Lane 1). In addition, the BRCA1-deficient MDA-MB-436 (Lane 3) and the BRCA1-competent SKBR-3 (Lane 4) were used as control for Western blots. NSB: non-specific protein band. (**B**) The clonogenic potential of HCC1937 ± BRCA1 cells (n = 6). (**C**,**D**) The aldehyde dehydrogenase activity measured by the AldeRed assay with representative flow cytometry data shown in (**C**) and quantitation in (**D**, n = 3). The DEAB treated cells were used as negative control. (E) Expression levels of three *ALDH1A* isoforms in HCC1937 ± BRCA1 cells measured by qRT-PCR (n = 3).

To examine the role of BRCA1 in the regulation of breast cancer cell stemness, we created a genetically matched pair of human breast cancer cell lines using the HCC1937 cell line derived from a Grade 3 primary ductal carcinoma with a loss-of-function mutation in the BRCA1 gene (insertion C at nucleotide 5382). The HCC1937BRCA1 cell line was generated by infection of retrovirus containing the full-length wild-type BRCA1 and the control line was made using the empty vector-containing viruses (Fig. 1A). Reconstitution with the wild-type BRCA1 significantly (p < 0.0001) suppressed the clonogenic potential of HCC1937 cells (Fig. 1B), an important characteristics of cancer stemness. We further determined the impact of BRCA1 on breast cancer stemness using the ALDH activity assay as a readout for the endogenous ALDH activities, a widely used functional assay of breast cancer stemness^28,29^. As shown in Fig. 1C,D, ectopic expression of BRCA1 in HCC1937 cells resulted in approximately 50% decrease of ALDH activities (p = 0.0032). ALDH1 has been shown to be the major contributor of ALDH activities in breast cancer cells^29–31^. Among its three isoforms, we found that *ALDH1A1* was significantly (p = 0.0012) down-regulated in BRCA1-reconstituted cells. In contrast, both *ALDH1A2* and *ALDH1A3* were expressed at much lower levels than *ALDH1A1* and their expression was not significantly affected by BRCA1.

**Figure 2:**
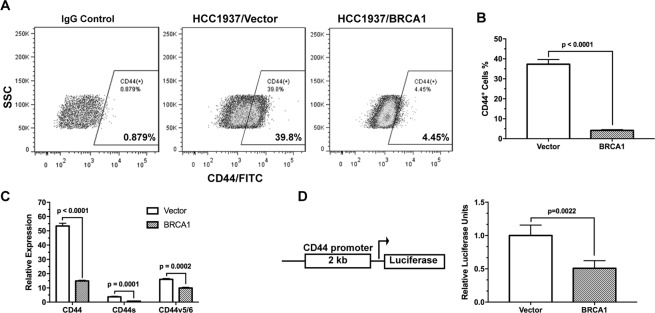
BRCA1 suppresses CD44 expression in breast cancer cells. The cell surface CD44 levels in HCC1937 ± BRCA1 cells were analyzed by flow cytometry with representative flow cytometry data shown in (**A**) and quantitation in (**B**, n = 3). (**C**) Expression levels of the total CD44 (including all isoforms/variants), CD44s, and the CD44v5/6 variant in HCC1937 ± BRCA1 cells measured by qRT-PCR (n = 3). (**D**) CD44 promoter activity in HCC1937 ± BRCA1 cells was measured using luciferase assay (n = 4).

The transmembrane glycoprotein CD44 is another commonly used cell surface marker of cancer stem cells^32,33^. Ectopic expression of BRCA1 in HCC1937 cells led to approximately a ten-fold decrease (p < 0.0001) in the CD44^+^ population (Fig. 2A,B). To examine the effects of BRCA1 in CD44 expression, we used a panel of qRT-PCR primers specific for all CD44 variants (CD44), the standard isoform (CD44s), and the CD44v5/6 variant. As shown in Fig. 2C, reconstitution of BRCA1 in HCC1937 resulted in significant suppression of the total *CD44* expression (p < 0.0001) including the CD44s (p = 0.0001) and CD44v5/6 (p = 0.0002) variants. Using a CD44 promoter-luciferase reporter containing the 2 kb CD44 promoter/enhancer region, we found that the transcription activity of the CD44 promoter is significantly (p = 0.0022) down-regulated in the BRCA1-reconstituted HCC1937 cells (Fig. 2D). It has been shown that BRCA1 can interact with the histone deacetylase complex^34^, thus affecting gene transcription. However, using chromatin immunoprecipitation, we found that ectopic expression of BRCA1 in HCC1937 cells did not reduce the level of histone H3 acetylation of *CD44* promoter, nor that of *ALDH1A* promoter. These observations suggest that BRCA1 is likely to be indirectly involved in down-regulation of the transcription of *CD44* and *ALDH1A*. Nonetheless, the results presented above indicate that restoration of BRCA1 in BRCA1-deficient breast cancer cells is sufficient to decrease their cancer stemness.

### Knockdown of BRCA1 enhances cancer stem cell-like characteristics

**Figure 3:**
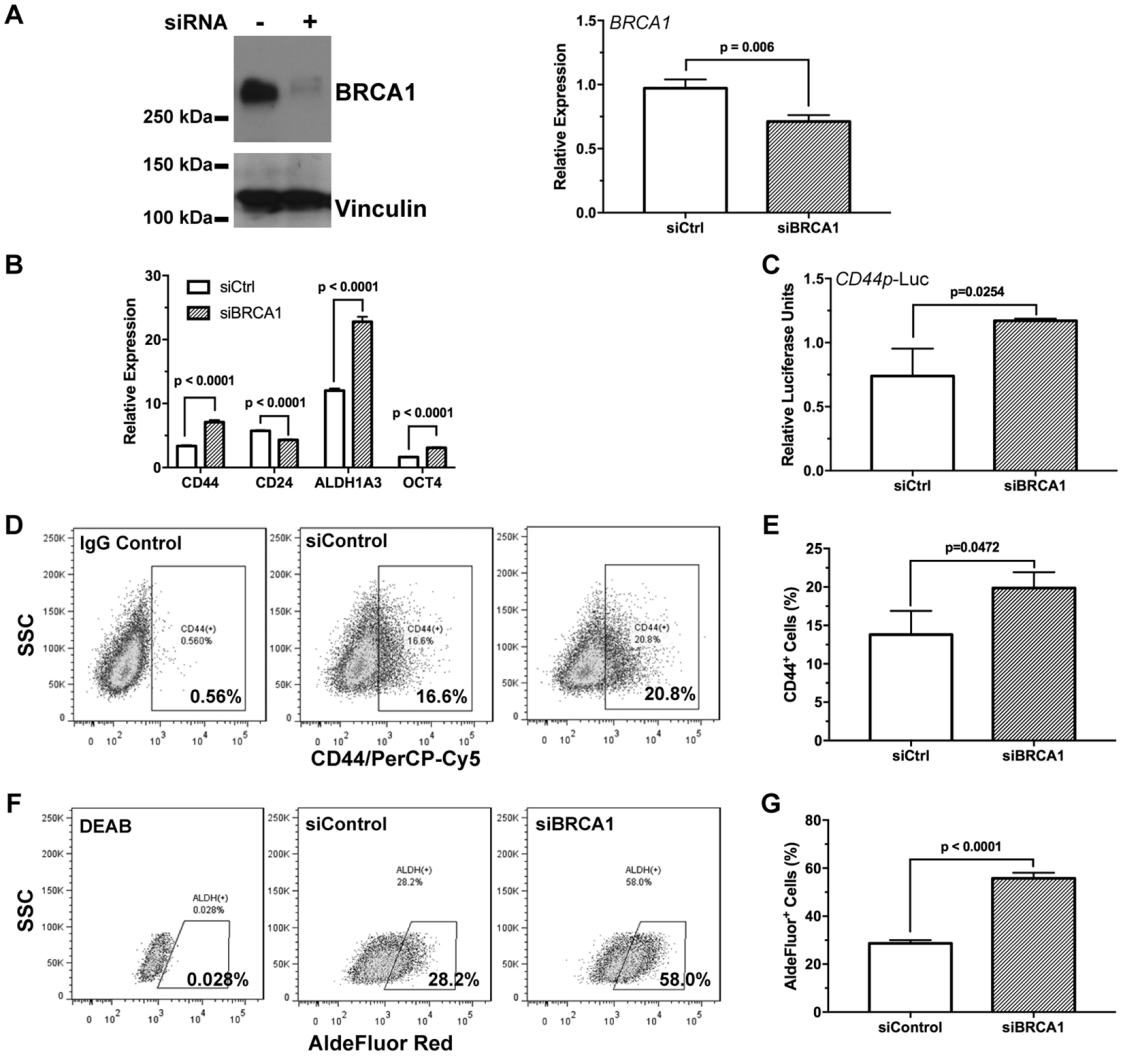
Down-regulation of BRCA1 promotes breast cancer stem cell characteristics. (**A**) BRCA1 was down-regulated by RNA inference in the BRCA1-competent SKBR-3 breast cancer cells, which was confirmed by Western blot and qRT-PCR (n = 3). (**B**) Expression of breast cancer stem cell-associated genes in SKBR3 ± siBRCA1 cells were measured by qRT-PCR (n = 3). (**C**) CD44 promoter activity in SKBR3 ± siBRCA1 cells was measured using luciferase assay (n = 3). The cell surface CD44 levels in SKBR3 ± siBRCA1 cells were analyzed by flow cytometry with representative flow cytometry data shown in (**D**) and quantitation in (**E**, n = 3). The ALDH activity in SKBR3 ± siBRCA1 cells were analyzed by flow cytometry with representative flow cytometry data shown in (**F**) and quantitation in (**G**, n = 3). The DEAB treated cells were used as negative control.

In addition to genetic mutations, BRCA1 expression can be down-regulated under stress conditions such as hypoxia^27,35^. To determine whether down-regulation of BRCA1 could affect breast cancer stem cell fate, we knocked-down BRCA1 in the BRCA1-competent human breast cancer cell line SKBR3 using RNA interference (Fig. 3A). Interestingly, siBRCA1 treatment resulted in significant increase in the expression of cancer stem cell-associated markers including CD44, ALDH1A3, and OCT4, while leading to down-regulation of CD24 (p < 0.0001 for all pair-wise comparisons, Fig. 3B). The transcription activity of the 2-kb CD44 promoter was moderately, but significantly (p = 0.0254), increased in the siBRCA1-treated SKBR3 cells (Fig. 3C). Consistent with the gene expression data, we found that the CD44^+^ population was significantly (p = 0.0472) increased upon siBRCA1 treatment (Fig. 3D,E). Knocking-down BRCA1 also significantly (p < 0.0001) increased ALDH^+^ population in the siBRCA1-treated tumor cells (Fig. 3F,G).

**Figure 4:**
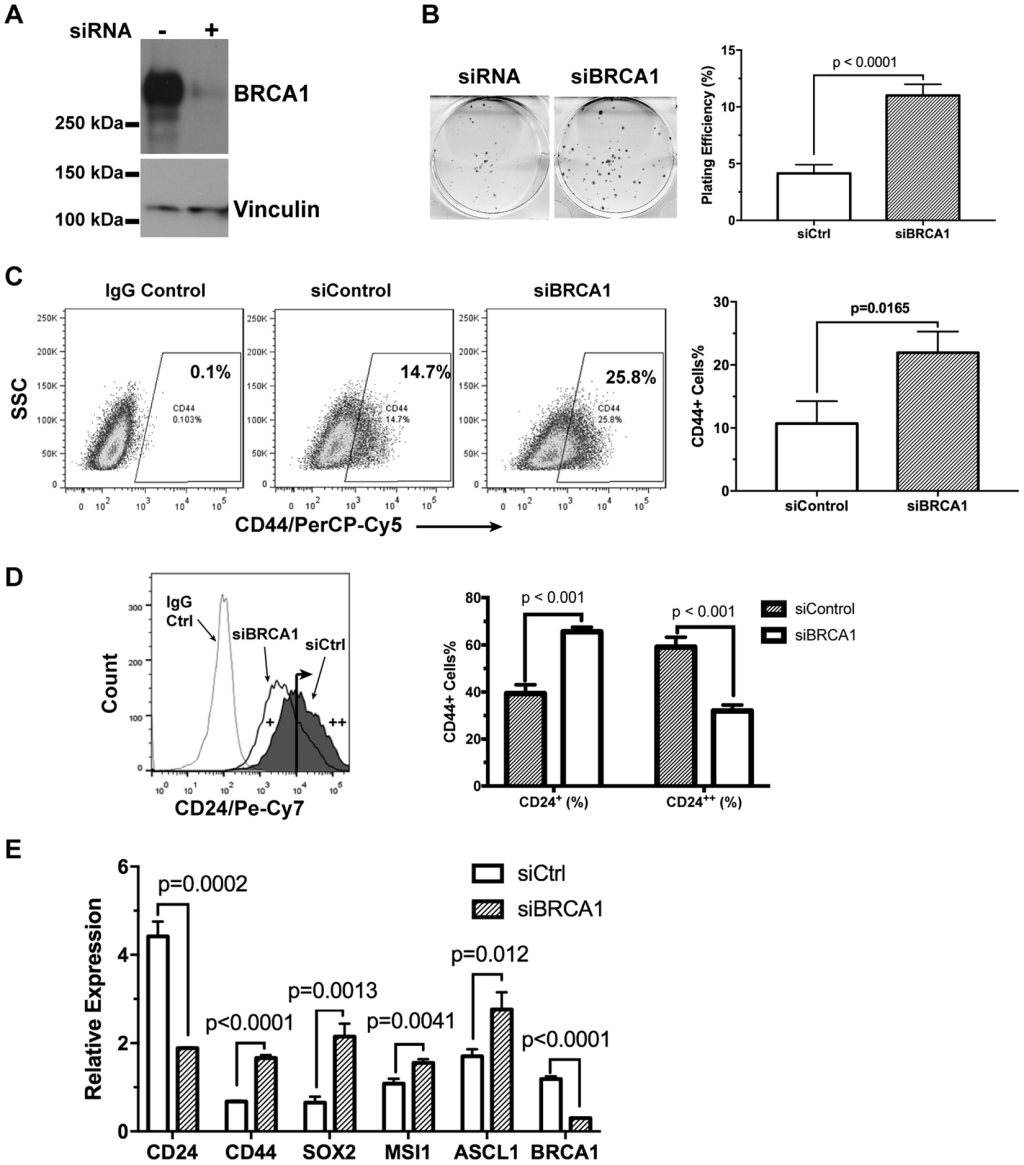
Down-regulation of BRCA1 promotes cancer cell stemness in neuroblastoma cells. BRCA1 was down-regulated by RNA inference in the SK-N-BE(2)C human neuroblastoma cells (Western blot shown in **A**). Effects of BRCA1 on neuroblastoma cell clonogenicity was measured by the clonogenic assay (**B**, n = 6). The cell surface levels of CD44 (**C**) and CD24 (**D**) in BE(2)C ± siBRCA1 cells were analyzed by flow cytometry (n = 3). Expression of neuroendocrine cancer stem cell-associated genes in BE(2)C ± siBRCA1 cells were measured by qRT-PCR (**E**, n = 3).

To determine whether BRCA1 plays a broad role in the regulation of cancer cell fate in general, we knocked-down BRCA1 expression in human neuroblastoma SK-N-BE(2)C cells (Fig. 4A). We found that siBRCA1-treatment resulted in a significant increase (p < 0.0001) of clonogenic growth of SK-N-BE(2)C cells (Fig. 4B), suggesting increased stemness. Consistently, the stemness-associated CD44^+^ population was significantly increased (p = 0.0165) in the siBRCA1-treated neuroblastoma cells (Fig. 4C). On the other hand, the cell population with high levels of the differentiation-associated CD24 (CD24^++^) were significantly decreased (p < 0.001), whereas the low expressor population (CD24^+^) were significantly increased (p < 0.001), as a consequence of BRCA1 knockdown (Fig. 4D). We further found that the stem cell-associated genes CD44, SOX2, MSI1, and ASCL1 were all upregulated, whereas CD24 was suppressed, upon siBRCA1 treatment (Fig. 4E). Collectively, these data have clearly demonstrated that down-regulation of BRCA1 can enhance cancer cell stemness in multiple tumor types.

### Hypoxia increases ALDH activities independent of BRCA1

**Figure 5:**
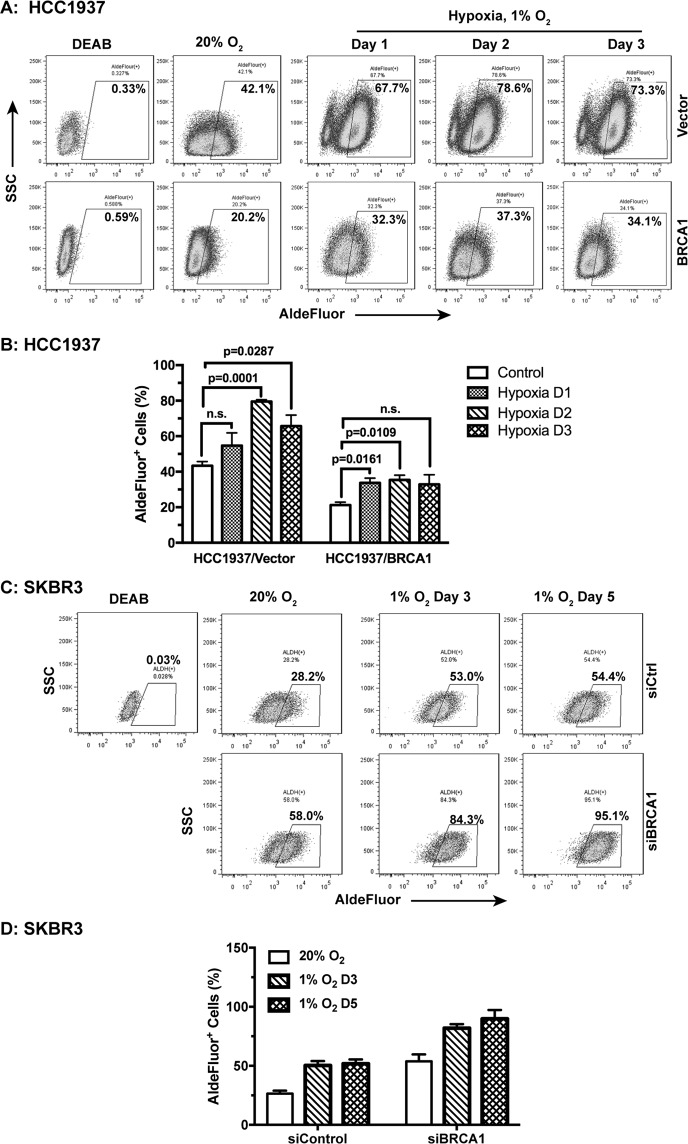
BRCA1 affects hypoxia-induced breast cancer cell stemness. HCC1937 ± BRCA1 cells were either maintained under ambient tissue culture conditions (20% O_2_) or under hypoxia (1% O_2_). Their ALDH activities were measured at the indicated time. The representative flow data are shown in (**A**) and quantitation in (**B**, n = 3). SKBR3 ± siBRCA1 cells were maintained under normoxia or hypoxia and their ALDH activities (ALDEFLUOR) were measured at the indicated time with the flow data shown in (**C**) and quantitation in (**D**, n = 2). The DEAB treated cells were used as negative control.

Hypoxia is a hallmark of tumor microenvironment (TME) in solid tumors. Tumor hypoxia is an independent prognostic factor for advanced disease progression and poor patient survival^36–38^. Studies from us and others have shown that hypoxia can facilitate the maintenance or enrichment of the cell population with stem cell characteristics^16,25,39–42^. We asked whether the BRCA1 status would affect breast cancer cell stemness under hypoxic conditions. Using the genetically matched pair of HCC1937 ± BRCA1 cells, we found that hypoxia significantly increased the ALDH^+^ populations of both parental (Vector) cells and the BRCA1-reconstituted HCC1937 cells in comparison to their respective normoxia (20% O_2_) controls (Fig. 5A,B). However, because BRCA1 strongly suppressed the ALDH activities, the hypoxia-increased ALDH^+^ population of HCC1937 + BRCA1 cells still did not reach the level of HCC1937/Vector control cells under normoxia (Fig. 5A,B). These results suggest that *BRCA1*, i.e. the genetic background, is a profoundly powerful determinant of breast cancer cell fate as compared to hypoxia, i.e. the microenvironment. Consistently, hypoxia strongly increased the ALDH^+^ populations of BRCA1-profiient SKBR3 (siCtrl) and the BRCA1-knocked-down SKBR3 (siBRCA1) cells (Fig. 5C). Nonetheless, much higher ALDH^+^ populations were observed in the siBRCA1-treated SKBR3, which further supports BRCA1 as an important regulator of breast cancer cell stemness.

### BRCA1-deficient breast cancer cells are less sensitive to HDAC inhibition

**Figure 6:**
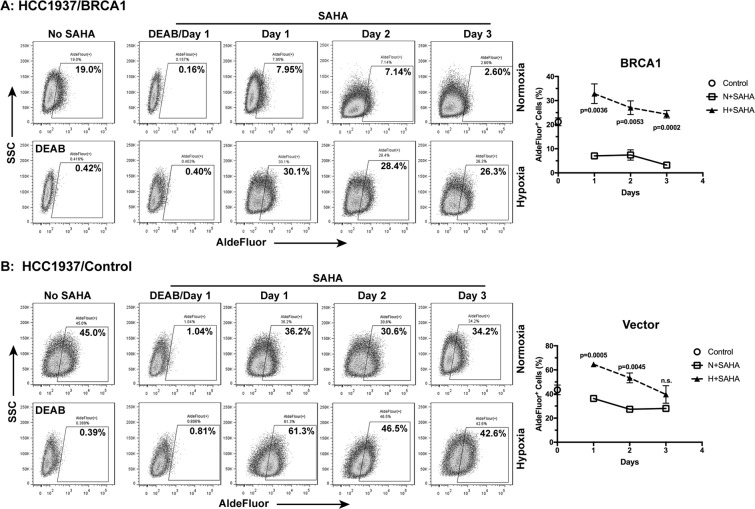
BRCA1 status and hypoxia determine breast cancer cell response to the histone deacetylase inhibitor SAHA. HCC1937 ± BRCA1 cells were treated with 1 μM SAHA under either normoxia or hypoxia. ALDH activities (ALDEFLUOR) were measured at the indicated time following incubation (n = 3). The DEAB treated cells were used as negative control.

In recent years, histone deacetylase (HDAC) inhibitors have emerged as an important class of anti-cancer drugs by inducing transcriptional and other epigenetic stresses in cancer cells^43,44^. In light of the role of BRCA1 in the regulation of both coding and non-coding RNAs^34,45^, we hypothesized that the status of BRCA1 would affect tumor cell response to histone deacetylases. We treated the genetically matched pair of parental (Vector) cells and the BRCA1-reconstituted HCC1937 breast cancer cells with SAHA^44^, a broad spectrum HDAC inhibitors and an FDA approved anti-cancer drug (Vorinostat), under normoxia and hypoxia, respectively. Using the ALDH activity assay, we found that SAHA strongly decreased ALDH activities in the BRCA1-reconstituted HCC1937 cells with the ALDH^+^ populations decreasing from >20% (untreated control, open circle on axis, Fig. 6A) to ≤5% under normoxia (open squares, Fig. 6A, p values shown in chart) without significantly affecting the BRCA1 protein levels (Supplementary Fig. 1). In contrast, SAHA induced moderate decrease of the ALDH^+^ HCC1937 parental (Vector) populations from >40% (untreated control, open circle on axis, Fig. 6B) to approximately 30% (open squares, Fig. 6B, p values shown in chart).

When treated with SAHA under the hypoxic condition, the ALDH^+^ HCC1937-BRCA1 population was both strongly and significantly increased as compared to their corresponding normoxia controls over 3 days of continuous drug treatment (closed triangle versus open squares, Fig. 6A). On the other hand, hypoxia was able to significantly increase and/or maintain the ALDH^+^ HCC1937-Vector population in the presence of SAHA for 2 days only (closed triangle versus open squares, Fig. 6B). The hypoxia effects diminished by the third day (Fig. 6B).

These results suggest that BRCA1-competent breast cancer cells are more sensitive, whereas BRCA1-deficient breast cancer cells are less sensitive, to SAHA-induced loss of stemness under normoxic conditions. Consistent with these findings, SAHA has been shown to induce morphological changes in human breast cancer cells to resemble morphology of differentiated cells with flat appearance and decreased nucleus-to-cytoplasm ratio^46^. Surprisingly, hypoxia has much stronger protective effects in the stemness of BRCA1-competent breast cancer cells. In light of the increased clinical efforts to use SAHA and other HDAC inhibitors for breast cancer therapy, our data suggest that both the BRCA1 status and the hypoxic tumor microenvironment are potential important parameters that may affect the clinical efficacy of HDAC inhibitors.

## Discussion

Cancer cell stemness is essential for enabling malignant progression and clonal evolution. The cell fate of cancer cells is likely to be determined by both cell-intrinsic pathways and by stress signals from tumor microenvironment. In this study, we have found that the tumor suppressor BRCA1, widely known as a key player in DNA repair, plays a critical role in the regulation of CSC-like characteristics. Ectopic expression of BRCA1 resulted in significant decrease of the CSC-like populations in human breast cancer cells whereas down-regulation of BRCA1 resulted in significant increase of the CSC-like population. Similar results are obtained from human neuroblastoma cells, suggesting BRCA1 has the potential to affect cell fate determinant in many tumor types. These results are consistent with the finding that BRCA1 is required for luminal differentiation of human mammary stem/progenitor cells^10^.

The exact mechanisms by which BRCA1 regulates cell fate remain to be delineated. BRCA1 is a multi-functional protein^47^. In addition to its classical role in homology-dependent DNA repair, BRCA1 has the potential to regulate gene transcription^47,48^ and to affect the epigenetic landscape via interacting with HDAC complexes^34^ and modulating mRNA expression^45^ among others. We have found that expression of the CSC-associated markers CD44 and ALDH1A is significantly increased in tumor cells with mutated or down-regulated BRCA1. However, the BRCA1 status did not significantly affect the level of histone H3 acetylation of *CD44* promoter and *ALDH1A* promoter, nor the activity of the *CD24* and *ALDH1* promoters (data not shown). We also found that reconstitution of BRCA1 resulted in significant increase of the breast cancer cell population expressing high levels of the differentiation-associated CD24 cell surface protein (Supplementary Fig. 2) without significantly affect *CD24* mRNA levels. These data suggest that transcription of *CD44*, *ALDH1A* and *CD24* may be differentially regulated by BRCA1-related epigenetic pathways, given the consensus finding that BRCA1 does not bind to DNA in a sequence-specific manner^47,48^. Nonetheless, our observations are consistent with previous findings that deletion or loss-of-function of *BRCA1* facilitates the expansion of mammary progenitor cells^49^ and promotes the development of basal-like mammary tumors^50,51^.

The interaction between BRCA1 and HDACs suggests that the BRCA1 status may affect tumor cell response to HDAC inhibitors. We have found that SAHA, an FDA-approved HDAC inhibitor, decreases the CSC-like population in both BRCA1-deficient and -competent breast cancer cells. Nonetheless, the BRCA1-reconstituted HCC1937 cells are much more sensitive to SAHA-induced loss of stemness than BRCA1-deficient HCC1937 parental cells. Given its ability to interact with HDACs^34^, BRCA1 might synergize with SAHA to create significant epigenetic stresses, which results in the loss of breast cancer cell stemness. Although the mechanisms remain to be examined, these data suggest that the BRCA1 status may determine tumor response to HDAC inhibitors.

Consistent with our previous findings, hypoxia, the hallmark of tumor microenvironment, significantly increases the CSC-like population in both BRCA1-deficient and -competent HCC1937 cells. However, the absolute increase in the CSC-like population in the BRCA1-reconstituted tumor cells under hypoxia remains small as compared to that in the BRCA1-mutated parental cells. These results suggest that BRCA1-deficiency may confer tumor cells with more efficient adaptability to changes in tumor microenvironment. However, both surprisingly and interestingly, hypoxia can significantly block SAHA-induced loss of stemness in the BRCA1-reconstituted HCC1937 cells, suggesting that hypoxia has the potential to disrupt the synergy between BRCA1 and SAHA in inducing breast cancer cell differentiation.

Currently, SAHA and other HDAC inhibitors have been actively tested in a large number of clinical trials involving breast cancers and many other cancers (ClinicalTrials.gov). In light of our findings, it is prudent to consider the BRCA1 status and tumor hypoxia as potentially important clinical parameters affecting the therapeutic efficacy of HDAC inhibitors.

## Methods

### Cell lines, cell culture, and hypoxia

HCC-1937 and SKBR3 human breast cancer cells (ATCC) were maintained in RPMI1640. SK-N-BE(2)C human neuroblastoma cells were maintained in MEM/F12 (1:1) supplemented with 10% fetal bovine serum. The experimental conditions for hypoxia were the same as described previously^52,53^. Briefly, the hypoxia culture media were supplemented with 25 mM HEPES at pH7.4 to strengthen the pH buffering capacity. Cells were incubated at 1% O_2_ in a hypoxia workstation (Ruskinn Invivo 400).

HCC-1937Vector and HCC-1937BRCA1 cell lines were generated by infection with retrovirus containing either pLZRS vector or pLZRS-BRCA1. For RNAi-mediated knockdown of BRCA1, SKBR3 cells were transiently transfected with Dharmacon human BRCA1 siRNA (BRCA1 ON-TARGET plus SMART pool) and negative control siRNA (ON-TARGET plus non-targeting pool) using the DharmaFECT™ transfection reagent according to manufacturer’s instruction.

### Chemical reagent

SAHA (suberoylanilide hydroxamic acid or Vorinostat, CAS #149647-78-9) was purchased from Cayman Chemicals. A 50 mM stock solution was prepared in DMSO. The working concentration was used at 1 μM.

### Analysis of cell surface markers by flow cytometry

Cell preparation, incubation with antibodies, and flow cytometry were performed using our previously published protocol^25^. Antibodies used for flow cytometry were purchased from eBiosciences (ThermoScientific): anti-CD24-PE-Cyanine 7 (antibody dilution of 1:25, catalogue #25-0247-42), anti-CD44-FITC (antibody dilution of 1:50, catalogue #11-0441-82), anti-CD44-PerCP-Cy5.5 (antibody dilution of 1:50, catalogue #45-0441-82). Isotype controls included mouse IgGκ-PE-Cyanine 7 (antibody dilution of 1:25, catalogue #25-4714-42), rat IgG2b-FITC (antibody dilution of 1:50, catalogue #11-4031-82), and rat IgG2b-PerCP-Cy5.5 (antibody dilution of 1:50, catalogue #45-4031-80). Cells were incubated with antibodies for 30 minutes on ice, filtered, and then analyzed on a BD LSR II flow cytometer. Singlets were gated for analysis, based on forward scatter (FSC) and side scatter (SSC) profiles. The instruments were calibrated daily. FACS data were analyzed using the FlowJo™ software.

### Aldehyde dehydrogenase (ALDH) activity assay

Cells were incubated using either the AldeRed ALDH Detection Assay kit (EMD Millipore, #SCR150) or the ALDEFLUOR kit (STEMCELL Technologies, #C01700) according to the manufacturer’s instructions.

### Clonogenic assay

The clonogenic assay is based on our previously published protocols^52,54^. Briefly, tumor cells were plated at 600 cells/well for MDA-MB-231 cells and at 1000 cells/well for SK-N-BE(2)C cells in 6-well plates and incubated for 10 to 14 days. Colonies were stained with Crystal Violet. Plating Efficiency (PE) = lnumbers of colonies (≥50 cells/colony) divided by numbers of cells plated × 100%.

### Western blot analysis

Whole cell lysates were prepared in RIPA buffer. Proteins were separated by SDS-PAGE under reducing conditions. BRCA1 protein was detected using mouse monoclonal anti-BRCA1 (SC-6954, 1:100, Santa Cruz Biotechnology). Protein bands were visualized using ECL substrates (BioRad, #170-5061) and imaged on Kodak X-OMAT 2000A.

### Real-time quantitative reverse transcription PCR (RT-qPCR)

Total cellular RNA was isolated with the TRizol reagent (Invitrogen). First-strand cDNA was synthesized using High Capacity cDNA Reverse Transcription Kit (Applied Biosystems). Levels of gene expression were performed by qRT-PCR on StepOne Plus (Applied Biosystems) using iTaq Universal SYBR Green Supermix (Bio-Rad) under the following conditions: initiation at 95 °C × 30 seconds, 40 cycles at 95 °C × 15 seconds, and 60 °C × 60 seconds. The house keeping gene HPRT was used as a control for normalization. Specificity of the primers (Table 1) was confirmed by a single peak on the dissociation curve.

**Table 1:** List of qRT-PCR primers.

Gene	Forward Primer	Reverse Primer
CD44 (all variants)	5′-TGCCGCTTTGCAGGTGTAT-3′	5′-GGCCTCCGTCCGAGAGA-3′
CD44s (NM_001001391 and NM_001202557)	5′-TACTGATGATGACGTGAGCA-3′	5′-GAATGTGTCTTGGTCTCTGGT-3′
CD44v5/6 (NM_001001389)	5′-GTAGACAGAAATGGCACCAC-3′	5′-CAGCTGTCCCTGTTGTCGAA-3′
BRCA1	5′-TCCTTCCTTGCAGGAAACCAGTCTC-3′	5′-TGAGGTTGTATCCGCTGCTTTGTCC-3′
MSI1	5′-CTCCAAAACAATTGACCCTAAGGT-3′	5′-GACAGCCCCCCCACAAAG-3′
ASCL1	5′-CACTGACTTTTGCTGCTGCTTCT-3′	5′-TGGCGCTCGCGTGTG-3′
ALDH1A1	5′-GCACGCCAGACTTACCTGTC-3′	5′-CCTCCTCAGTTGCAGGATTAAAG-3′
ALDH1A2	5′-AGGCCCTCCTCGCTCAC-3′	5′-TCTGCCCCAGAATGAGCTC-3′
ALDH1A3	5′-GCATGAGCCCATTGGTGTCT-3′	5′-CGCAGGCTTCAGGACCAT-3′
OCT4	5′-GAGAACCGAGTGAGAGGCAACC-3′	5′-CATAGTCGCTGCTTGATCGCTTG-3′
CD24	5′-AAACAACAACTGGAACTTCAAGTAACTC-3′	5′-GGTGGTGGCATTAGTTGGATTT-3′
SOX2	5′-TGCGAGCGCTGCACAT-3′	5′-GCAGCGTGTACTTATCCTTCTTCA-3′
HPRT	5′-TATGGCGACCCGCAGCCCT-3′	5′-GGTGGTGGCATTAGTTGGATTT-3′

### CD44 promoter activity luciferase assay

The reporter CD44P pGL3, a gift from Dr. Robert Weinberg (Addgene plasmid # 19122), contains 2021 bp of the human CD44 promoter/enhancer upstream of the translation initiation site. CD44P pGL3 reporter plasmid was co-transfected with 0.2 μg Renilla Luciferase plasmid DNA (Promega, Mannheim, Germany) as a control for transfection efficiency. After 48 hr incubation, transfected cells were lysed using the Dual-Luciferase Reporter Assay System (Promega). Promoter-driven luciferase activity was measured on the Llumat LB 9507 machine (EG&G Berthold Co.) and normalized to the Renilla Luciferase activity. Each experiment was carried out in triplicates and repeated three times.

### Statistics

Two group comparisons were analyzed by 2-tailed Student’s *t*-test (GraphPad Prism 7). Significant difference was declared if p < 0.05.

## Supplementary information

Supplementary Information

## Data Availability

Data presented in this manuscript will be made available upon request.
