# Supplementary material for: BRCA1 regulates the cancer stem cell fate of breast cancer cells in the context of hypoxia and histone deacetylase inhibitors
Source: Sci Rep. 2019 Jul 4;9:9702. doi: 10.1038/s41598-019-46210-y (PMC6609720; doi:10.1038/s41598-019-46210-y)

Supplementary Information

BRCA1 regulates the cancer stem cell fate of breast cancer cells in the context of hypoxia and histone deacetylase inhibitors.

Hoon Kim, Qun Lin, and Zhong Yun*

Department of Therapeutic Radiology, Yale School of Medicine, New Haven, CT 06510, USA

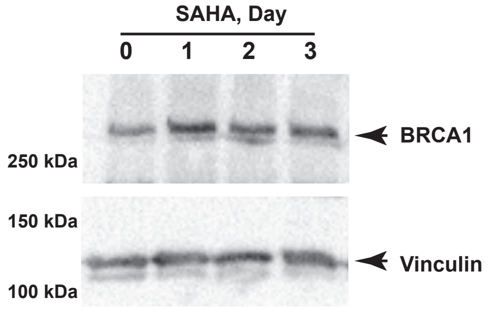

Supplementary Figure 1. BRCA1 protein expression is not affected by the HDAC inhibitor SAHA. BRCA1-reconstituted HCC1937 cells were incubated with 1 μM SAHA under ambient conditions. Whole cell lysates were collected daily for three days with vehicle control as Day 0. BRCA1 was examined by Western blot with vinculin as a control.

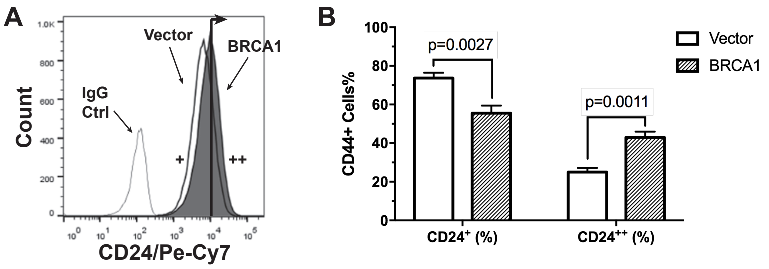

Supplementary Figure 2. Reconstitution of BRCA1 expression in HCC1937 cell increases the $CD24^{++}$ population. Cell surface levels of CD24 were analyzed by flow cytometry. The total $CD24^{+}$ populations were further separated into$CD24^{+}$ and $CD24^{++}$ populations by the vertical line and an arrow (A). The relative distribution of these two populations were quantified from three independent experiments (B).

Original Western blots

Fig. 1A

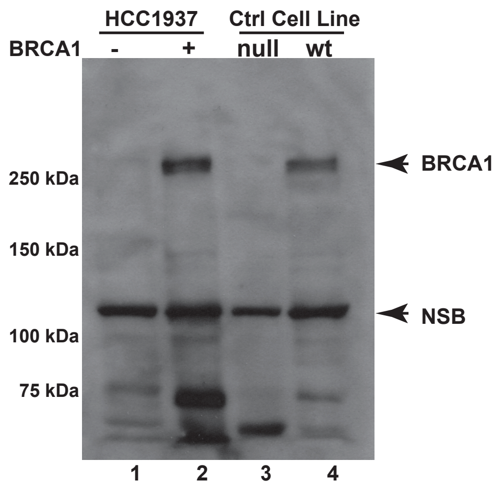

Control Cell Lines

Lane 3: MDA-MB-436: BRCA1-null

Lane 4: SKBR3: BRCA1 wild-type (wt)

Fig. 3A

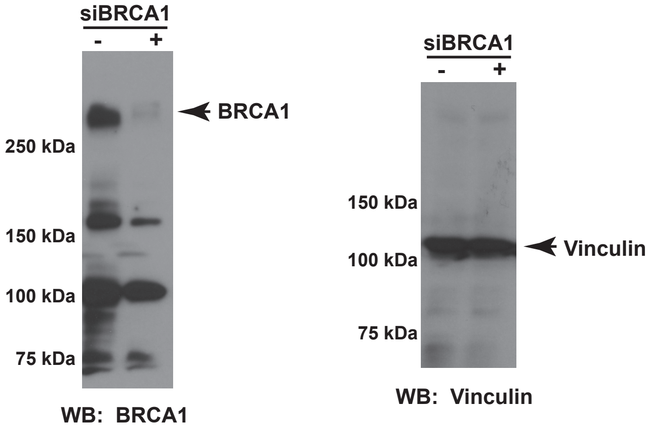

Fig. 4A

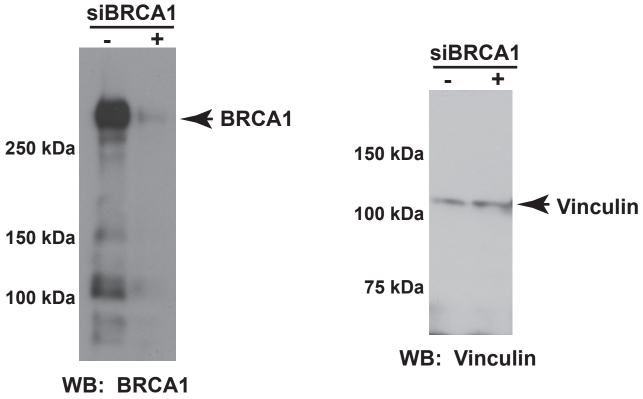

Supplement: Supplementary file 1 — Supplementary Information [file 41598_2019_46210_MOESM1_ESM.pdf]
